# Astrovirus in White-Tailed Deer, United States, 2018

**DOI:** 10.3201/eid2602.190878

**Published:** 2020-02

**Authors:** Leyi Wang, Huigang Shen, Ying Zheng, Loni Schumacher, Ganwu Li

**Affiliations:** University of Illinois, Urbana, Illinois, USA (L. Wang);; Iowa State University, Ames, Iowa, USA (H. Shen, Y. Zheng, L. Schumacher, G. Li);; Chinese Academy of Agricultural Sciences, Harbin, China (G. Li)

**Keywords:** white-tailed deer, astrovirus, respiratory diseases, viruses, United States, ORF1a, ORF1b, capsid, bacteria, next-generation sequencing, co-infection, phylogenetic analysis, bovine astroviruses, recombinant virus

## Abstract

We report the identification of astrovirus WI65268 in a white-tailed deer with respiratory disease in the United States in 2018. This virus is a recombinant of Kagoshima1-7 and Kagoshima2-3-2 (both bovine astroviruses from Japan) and was characterized as a potential new genotype. Further surveillance of deer might help identify related isolates.

Astrovirus is a positive-sense, single-stranded RNA virus first identified in feces of children with gastroenteritis in 1975. Since then, astrovirus has been found in a wide variety of mammals and birds (*1*). The family *Astroviridae* comprises 2 genera, *Mamastrovirus* and *Avastrovirus*, and classification is based on host origin. Astroviruses cause diarrhea and neurologic diseases in mammals and a spectrum of diseases, including diarrhea, hepatitis, and nephritis, in birds (*2*). Astrovirus is associated with respiratory disease in humans, cattle, and pigs (*3*–*5*) and has also been found in fecal samples from roe deer with gastrointestinal illness in Denmark (*6*). Whether astrovirus circulates in other species of deer remains unclear.

In September 2018, the Veterinary Diagnostic Laboratory at Iowa State University (Ames, Iowa, USA) received 5 sets of tissue samples collected from deer of the same farm for identification of the infectious cause of death of 5 male white-tailed deer 8–14 weeks of age. The pen-raised deer experienced pneumonia and sudden death. Postmortem examinations showed pleural fluid in the lungs, pneumonia, and purple-mottled lungs. Histopathologic observations revealed that 3 deer had necrotizing bronchopneumonia, and 2 had interstitial pneumonia. 

Although different combinations of the bacterial pathogens *Bibersteinia trehalosi*, *Tureperella pyogenes*, *Fusobacterium necrophorum*, and *Pasteurella multocida* were found in all cases, an underlying viral cause could not be excluded. Therefore, we used next-generation sequencing, first with pooled lung samples and then with individual lung samples, using Nextera XT DNA Library Preparation Kit with the MiSeq platform and MiSeq Reagent Kit v2 (Illumina, https://www.illumina.com). A bioinformatic analysis indicated the presence of an astrovirus along with the bacteria. The complete genome sequence (6,246 nt) of this astrovirus (WI65268; GenBank accession no. MN087316) was found in the pooled lung tissue sample and 1 lung tissue sample, and partial genomes were found in the other 4 lung samples. A complete-genome comparison revealed that BoAstV/JPN/Ishikawa24-6/2013 (bovine isolate from Japan) had the highest identity (60.9%) to WI65268. Further nucleotide sequence analysis revealed that WI65268 had a similar genome organization as other astroviruses (Appendix Figure 1).

Sequence comparisons of the amino acid sequences of the 3 open reading frames (ORFs) showed that WI65268 was closely related to 4 bovine astroviruses from Asia: B18 (ORF1a 71.9% sequence identity), Kagoshima1-7 and B76-2 (ORF1b 87.8% sequence identity), and Hokkaido11-55 (ORF2 46.8% sequence identity, distance value 0.479) (Appendix Table). In contrast, WI65268 showed low amino acid sequence identities to US bovine strain BSRI-1 for all 3 ORFs (ORF1a 37.0%, ORF1b 68.3%, ORF2 38.8%) (Appendix Table). The 2 available astrovirus sequences from roe deer (GenBank accession nos. HM447045 and HM447046) from Europe comprised only partial genomic sequences. WI65268 had low identities (34.0% HM447045 and 34.4% HM447046) and pairwise distances (0.787 HM447045 and 0.813 HM447046) to these isolates. On the basis of the International Committee on Taxonomy of Viruses p-distance criteria (new genotypes are assigned at a value of >0.378) (*7*), WI65268 represents a novel astrovirus genotype.

Phylogenetic analysis of the complete genome showed that WI65268 is distantly related to other bovine, dromedary, takin, and yak strains (Appendix Figure 2). In phylogenetic analyses of ORF1a and ORF1b protein sequences, WI65268 clustered with bovine, yak, and takin astrovirus isolates from Asia (Appendix Figure 3). However, in an analysis of ORF2 (capsid) protein, WI65268 clustered with 2 bovine isolates from Japan and was distantly related to the cluster formed by the bovine, yak, and takin isolates from Asia (Figure), strongly indicating that WI65268 is a recombinant. We used Recombination Detection Program 5 (http://web.cbio.uct.ac.za/~darren/rdp.html) to confirm that WI65268 was a recombinant and characterize the recombination event (Kagoshima1-7 at 1–5,031 and 5,651–7,967 and Kagoshima2-3-2 at 5,032–5,650; Appendix Figure 4). Reverse transcription PCR and sequencing results confirmed that sequences at the 2 junctional sites were the same as those found by next-generation sequencing.

**Figure:**
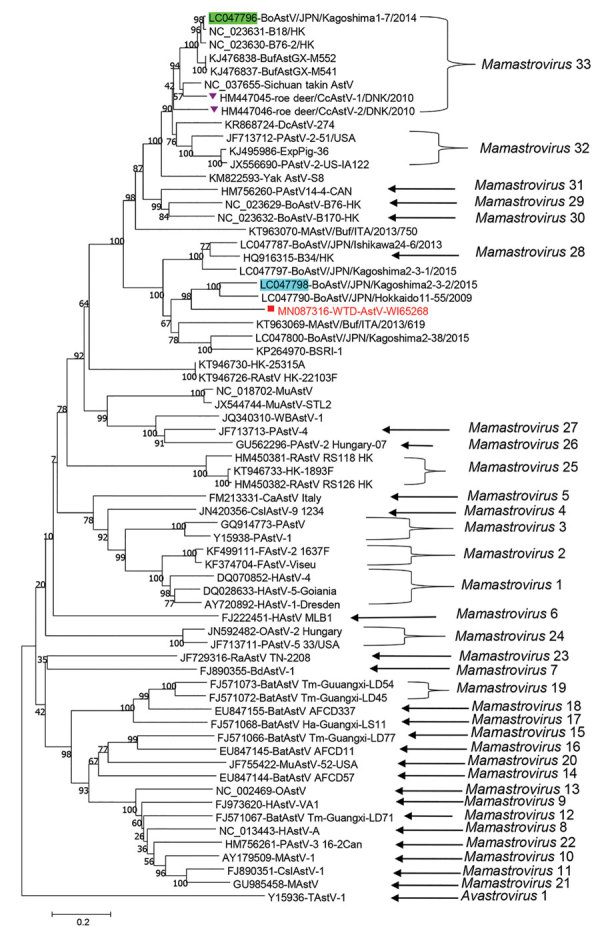
Phylogenetic analysis of amino acid sequence of open reading frame 2 of WTD-AstV WI65268 from deer in the United States, 2018 (red square), and potential parent viruses, including Kagoshima1-7 (green highlight), Kagoshima2-3-2 (blue highlight), and CcAstVs (purple triangles). Genus type is provided for viruses where that information was known. GenBank accession numbers are indicated, and bootstrap values are provided at nodes. Scale bar indicates amino acid changes per site. AstV, astrovirus; BdAstV, bottlenose dolphin astrovirus; BoAstV, bovine astrovirus; BufAst, water buffalo astrovirus; CaAstV, canine astrovirus; CcAstV, *Capreolus capreolus* astrovirus; CsIAstV, California sea lion astrovirus; DcAstV, dromedary camel astrovirus; FAstV, feline astrovirus; HAstV, human astrovirus; MAstV, mink astrovirus; MuAstV, murine astrovirus; OAstV, ovine astrovirus; PAstV, porcine astrovirus; RAstV, rat astrovirus; RaAstV, rabbit astrovirus; TAstV, turkey astrovirus; WBAstV, wild boar astrovirus; WTD, white-tailed deer.

Pathogens causing respiratory disease in domesticated animals, such as cattle and pigs, are relatively well studied. However, pathogens causing these diseases in wildlife animals, such as deer, are not well characterized. In this study, the new astrovirus we found or the bacterial pathogens could have contributed to the respiratory disease observed. Whether astrovirus plays a major or just synergistic role in respiratory disease in deer should be explored further.

Astrovirus was previously identified in roe deer with gastrointestinal illness in Europe and found to be closely related to bovine astrovirus isolates from Hong Kong, China, of the same genus (*Mamastrovirus 33*) (*7*,*8*). WI65268 was also closely related to bovine isolates from Japan but distantly related to roe deer and Hong Kong bovine astrovirus isolates. An additional analysis of genetic distances of related isolates on the basis of ORF2 tentatively classified WI65268 as a novel species (Appendix Table).

Determining the evolution of WI65268 any further is difficult without further epidemiologic data. Bovine or bovida astroviruses might be able to cross species barriers and replicate in deer, as suggested in a previous study (*9*), in which a bovine astrovirus isolate clustered with a porcine astrovirus type 5 instead of other bovine astroviruses. Further surveillance of white-tailed deer for astrovirus is needed for field monitoring.

Appendix More information about astrovirus in white-tailed deer, United States, 2018.
