# Supplementary material for: Astrovirus in White-Tailed Deer, United States, 2018
Source: Emerg Infect Dis. 2020 Feb;26(2):374–6. doi: 10.3201/eid2602.190878 (PMC6986847; doi:10.3201/eid2602.190878)
Supplement: Appendix — More information about astrovirus in white-tailed deer, United States, 2018. [file 19-0878-Techapp-s1.pdf]

Article DOI: https://doi.org/10.3201/eid2602.190878

Astrovirus in White-Tailed Deer, United States, 2018

Appendix

Targeted Amplification

PCR products of 5′ end 597 bp and 3′ end 576 bp were amplified using primers 5′ end forward primer TAGTTGAGATTGAGCGTAATAAATA and reverse primer CTCAAGCCATATGTTAAGCTTAAGCA and 3′ end forward primer GTCGCACAGTTTATGTGTGTTG and reverse primer CCCTTCACCTATGCAATCAAATCACAA, respectively, and sequenced on MiSeq. The sequence analysis showed that both ends of sequences are exactly same as the original complete sequence obtained by metagenomic sequencing. The junction sites between open reading frame (ORF) 1a and ORF1b as well as between ORF1b and ORF2 were amplified using primer sets DeerAstV-ORF1ab forward and reverse primers CAATATTTAGACCGGGACTATGATGC and CAAAAGCGGGATGGCTCGGCA, respectively, and DeerAstV-ORF12 forward and reverse primers GTGGCTTTACAGTTGGGAACAAC and TATTTGACGCTGAGACGGAGCAA, respectively. The reverse transcription PCR and sequencing results revealed that the sequences at the 2 junctional sites (ORF1a-1b, ORF1–2) were same as those from next-generation sequencing, which confirmed that there was only one strain in the sample instead of 2 distinct strains.

Detailed Genomic Information of WTD-AstV-WI65268

WTD-AstV-WI65268 has three complete overlapping ORFs: ORF1a at position 25–2478 nt encoding 817aa, ORF1b at position 2433–3941 nt encoding 502aa, and ORF2 at position 3886–6168 encoding 760aa. WTD-AstV-WI65268 had the conserved ‘slipper heptamer’ AAAAAAC sequence near the 3′ end of the ORF1a for inducing ribosomal frameshift during polyprotein nsp1ab translation. The highly conserved promoter sequence for subgenomic RNA

synthesis among mammalian AstVs UUUGGAGNGGNGGACCNAAN11AUGNC was present at the start of ORF2 in the WTD-AstV-WI65268.

Recombination in Astrovirus (AstV)

In addition to genomic mutation, recombination has been reported in different viral families. Recombination in ORF2 of bovine AstV was previously reported (*1*). WTD AstV was found to a recombinant between 2 Japan bovine strains with the recombination junction in ORF2. Recombination in ORF2 will allow generation of divergent viral progeny to enhance viral immune evasion function.

Reference

1. Tse H, Chan WM, Tsoi HW, Fan RY, Lau CC, Lau SK, et al. Rediscovery and genomic characterization of bovine astroviruses. J Gen Virol. 2011;92:1888–98. PubMed https://doi.org/10.1099/vir.0.030817-0

Appendix Table. Comparison of amino acid sequence identity of ORF2, ORF1a, ORF1b and Pairwise Distance of WTD-AstV-WI65268 to other strains in GenBank*

Astrovirus strain information	ORF2	ORF1a	ORF1b	Distance†
LC047790-BoAstV/JPN/Hokkaido11–55/2009	46.8	39.9	68.7	0.479
LC047798-BoAstV/JPN/Kagoshima2–3-2/2015	46.2	40.8	68.5	0.487
LC047787-BoAstV/JPN/Ishikawa24–6/2013	44.8	70.7	87.2	0.582
LC047797-BoAstV/JPN/Kagoshima2–3-1/2015	44.5	70.8	87.6	0.595
HQ916315-B34/HK	42.0	-	87.4	0.658
KT963069-MAstV/Buf/ITA/2013/619	40.2	-	-	0.644
LC047800-BoAstV/JPN/Kagoshima2–38/2015	38.8	39.6	68.3	0.658
KP264970-BSRI-1	38.8	37.0	68.3	0.626
NC_023629-BAstV-B76-HK	36.8	67.6	84.6	0.776
KJ476837-BufAstGX-M541	36.5	-	-	0.813
LC047796-BoAstV/JPN/Kagoshima1–7/2014	36.4	71.6	87.8	0.803
KJ476838-BufAstGX-M552	36.2	-	-	0.813
NC_023631-BAstV-B18 HK	36.0	71.9	87.2	0.803
NC_023630-B76–2/HK	35.8	70.7	87.8	0.808
NC_023632-BAstV-B170-HK	35.8	67.4	84.4	0.792
KM822593-YakAstV-S8	354	71.0	87.4	0.803
HM447046-deer/CcAstV-2/DNK/2010	34.4	-	-	0.813
HM447045-deer/CcAstV-1/DNK/2010	34.0	-	-	0.787
KR868724-DcAstV-274	33.4	64.5	82.6	0.776
JX556690-PAstV-2-U.S.-IA122	32.8	65.5	82.3	0.852
JF713712-PAstV-2–51/USA	32.3	66.8	81.8	0.835
KJ495986-ExpPig-36	31.3	66.7	80.5	0.835
HM756260-PAstV14–4-CAN	31.2	-	-	0.881
KT963070-MAstV/Buf/ITA/2013/750	30.4	-	-	0.922
KT946730-HK-25315A	24.5	30.4	58.6	0.978
KT946726-RAstV HK-22103F	24.5	8.6	58.4	0.978
JF713713-PAstV-4	23.9	29.4	58.0	0.922
NC_018702-MuAstV	23.2	27.2	57.1	0.991
JX544744-MuAstV-STL2	23.2	26.4	56.9	0.985
JQ340310-WBAstV-1	22.3	27.9	54.9	0.991
GU562296-PAstV-2 Hungary-07	21.3	-	-	0.991
KT946733-HK-1893F	21.2	25.5	57.5	1.052
HM450382-RAstV RS126 HK	21.0	-	57.5	1.052
AY720892-HAstV-1-Dresden	20.9	18.2	57.8	1.087

Astrovirus strain information	ORF2	ORF1a	ORF1b	Distance†
HM450381-RAstV RS118 HK	20.8	-	56.9	1.080
JF713711-PAstV-5 33/USA	20.6	18.3	46.4	1.184
DQ028633-HAstV-5-Goiania	20.3	18.8	56.0	1.094
JN592482-OAstV-2 Hungary	20.2	-	-	1.208
KF374704-FAstV-Viseu	20.1	19.4	56.1	1.072
KF499111-FAstV-2 1637F	20.0	18.9	55.2	1.087
DQ070852-HAstV-4	19.7	18.6	57.6	1.101
FM213331-CaAstV Italy	19.5	-	-	1.101
FJ890355-BdAstV-1	19.4	-	-	1.116
JN420356-CslAstV-9 1234	19.2	19.5	53.5	1.130
Y15938-PAstV-1	18.6	-	-	1.200
GQ914773-PAstV	17.7	-	-	1.249
JF729316-RaAstV TN-2208	17.6	18.4	55.1	1.161
FJ222451-HAstV MLB1	16.9	20.7	51.1	1.301
FJ571068-BatAstV Ha-Guangxi-LS11	16.7	-	-	1.249
EU847144-BatAstV AFCD57	16.7	-	-	1.249
FJ571066-BatAstV Tm-Guangxi-LD77	16.4	-	-	1.292
FJ571072-BatAstV Tm-Guangxi-LD45	16.3	-	-	1.208
FJ973620-HAstV-VA1	15.9	20.0	48.2	1.310
FJ571073-BatAstV Tm-Guuangxi-LD54	15.4	-	-	1.249
FJ890351-CslAstV-1	15.3	-	-	1.328
NC_013443-HMOAstV-A	15.0	19.4	48.1	1.346
FJ571067-BatAstV Tm-Guangxi-LD71	14.9	-	-	1.365
EU847155-BatAstV AFCD337	14.9	-	-	1.283
AY179509-MAstV-1	14.9	18.6	50.8	1.337
NC_002469-OAstV	14.5	19.5	48.9	1.374
JF755422-MouseAstV-52-USA	14.5	15.5	47.6	1.384
HM756261-PoAstV-3_16–2Can	14.4	-	-	1.346
EU847145-BatAstV AFCD11	14.3	-	-	1.337
GU985458-SMS-AstV	13.9	-	-	1.346
Y15936-TAstV-1	12.8	11.3	36.4	1.540

*ORF, open reading frame.
†Pairwise distance was calculated using the MEGA version 7.0.26

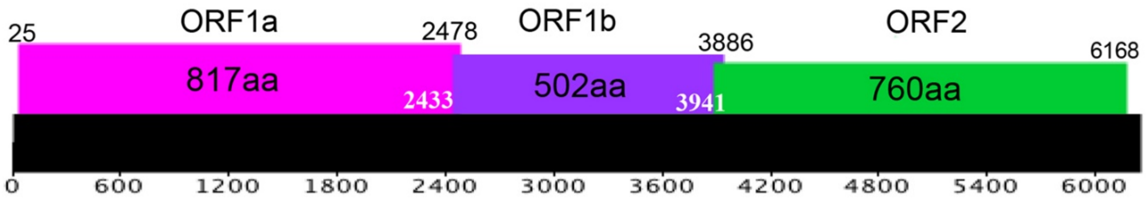

Appendix Figure 1. Schematic diagram of WTD-AstV-WI65268 genome. ORF, open reading frame.

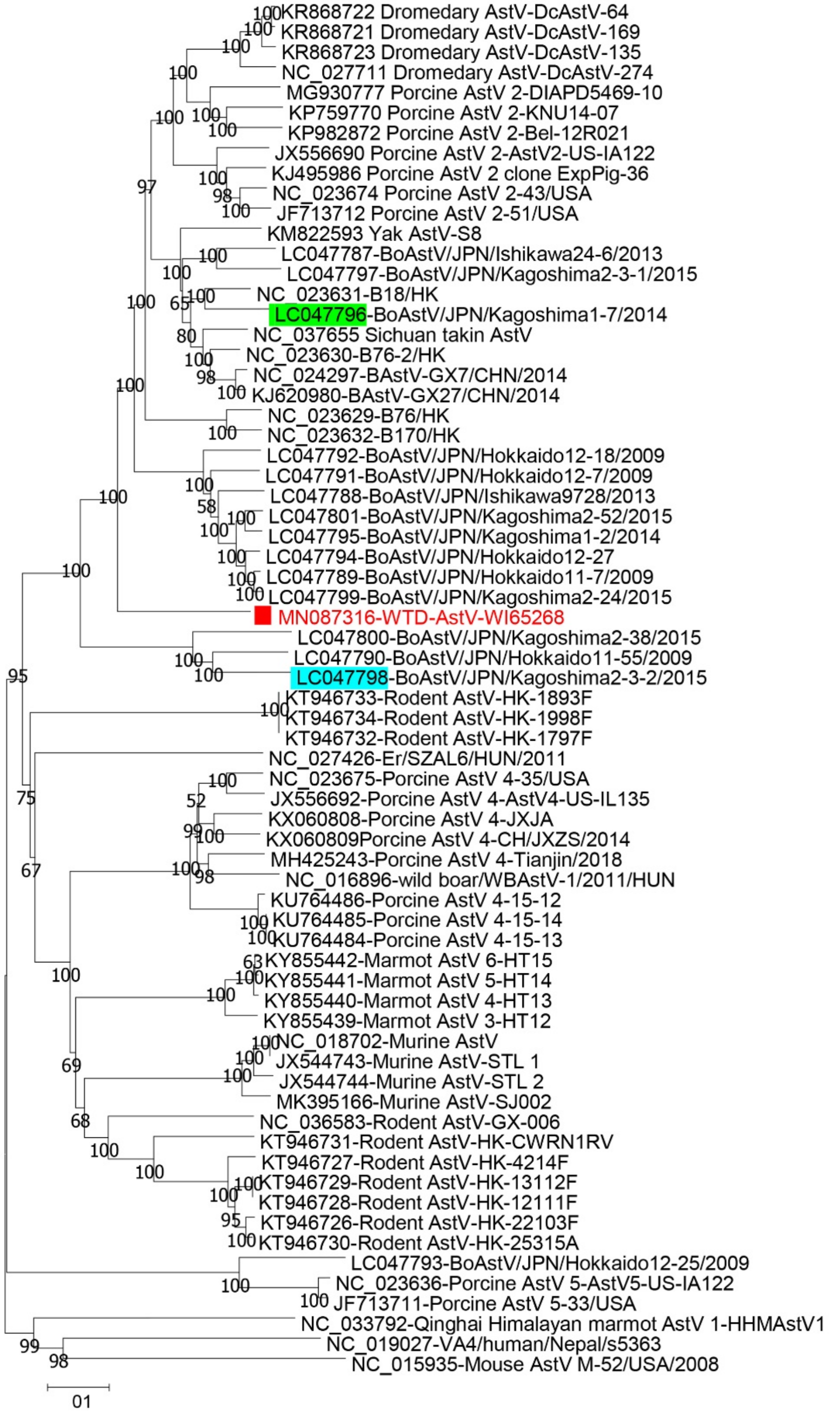

Appendix Figure 2. Phylogenetic tree analysis of complete genome sequences of astrovirus including WTD-AstV-WI65268 (indicated with a red square) and its potential parent virus strains Kagoshima1–7 and Kagoshima2–3-2 were marked with green and turquoise colors. The sequences acquired from GenBank were labeled with their accession numbers. Bootstrap values are indicated at nodes. Scale bar indicates 0.1 nucleotide changes per site.

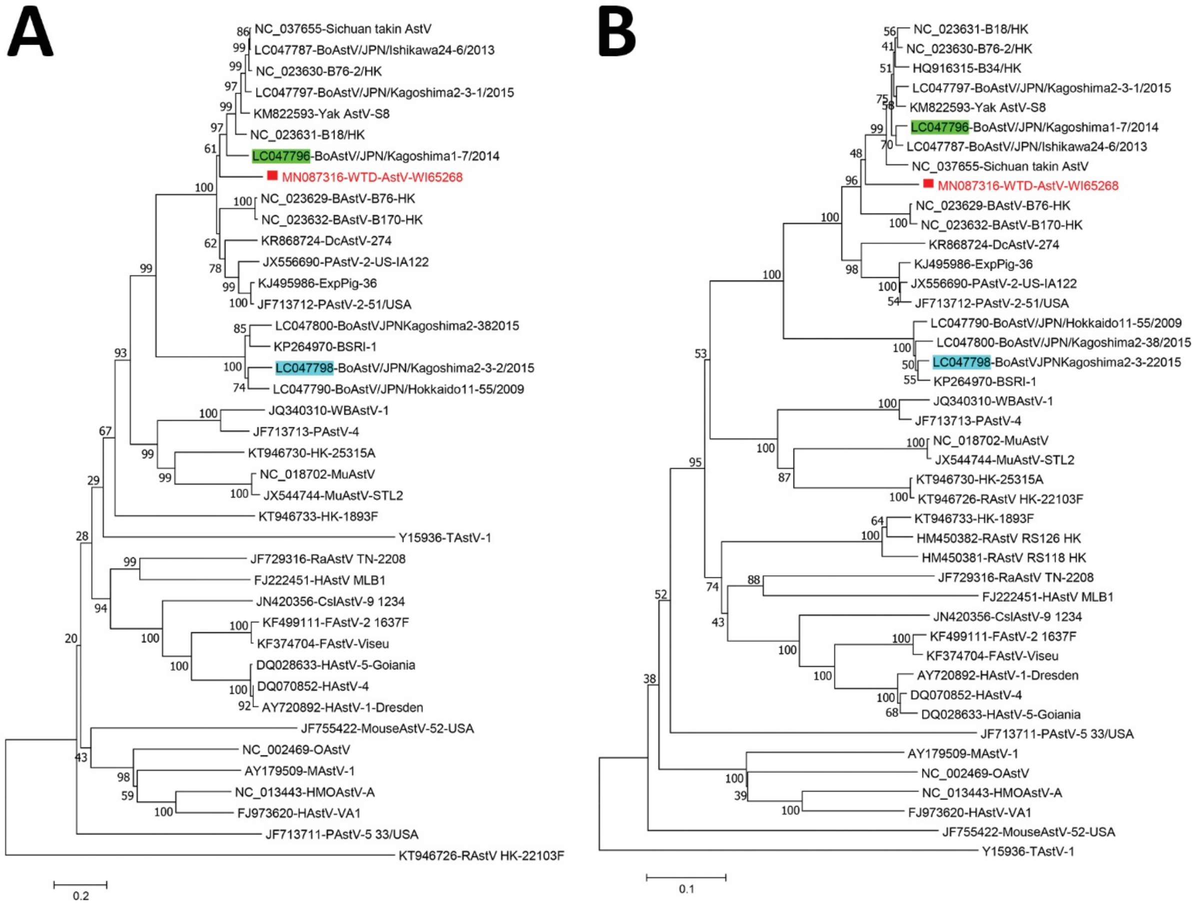

Appendix Figure 3. Phylogenetic analyses of amino acid sequences of open reading frame 1a (A) and 1b (B) of astrovirus WI65268, United States, 2018 (red square), and potential parent viruses, including Kagoshima1-7 (green highlight) and Kagoshima2-3-2 (turquoise highlight). GenBank accession numbers of sequences are provided. Scale bar indicates 0.2 and 0.1 amino acid changes per residue site in panel A and B, respectively.

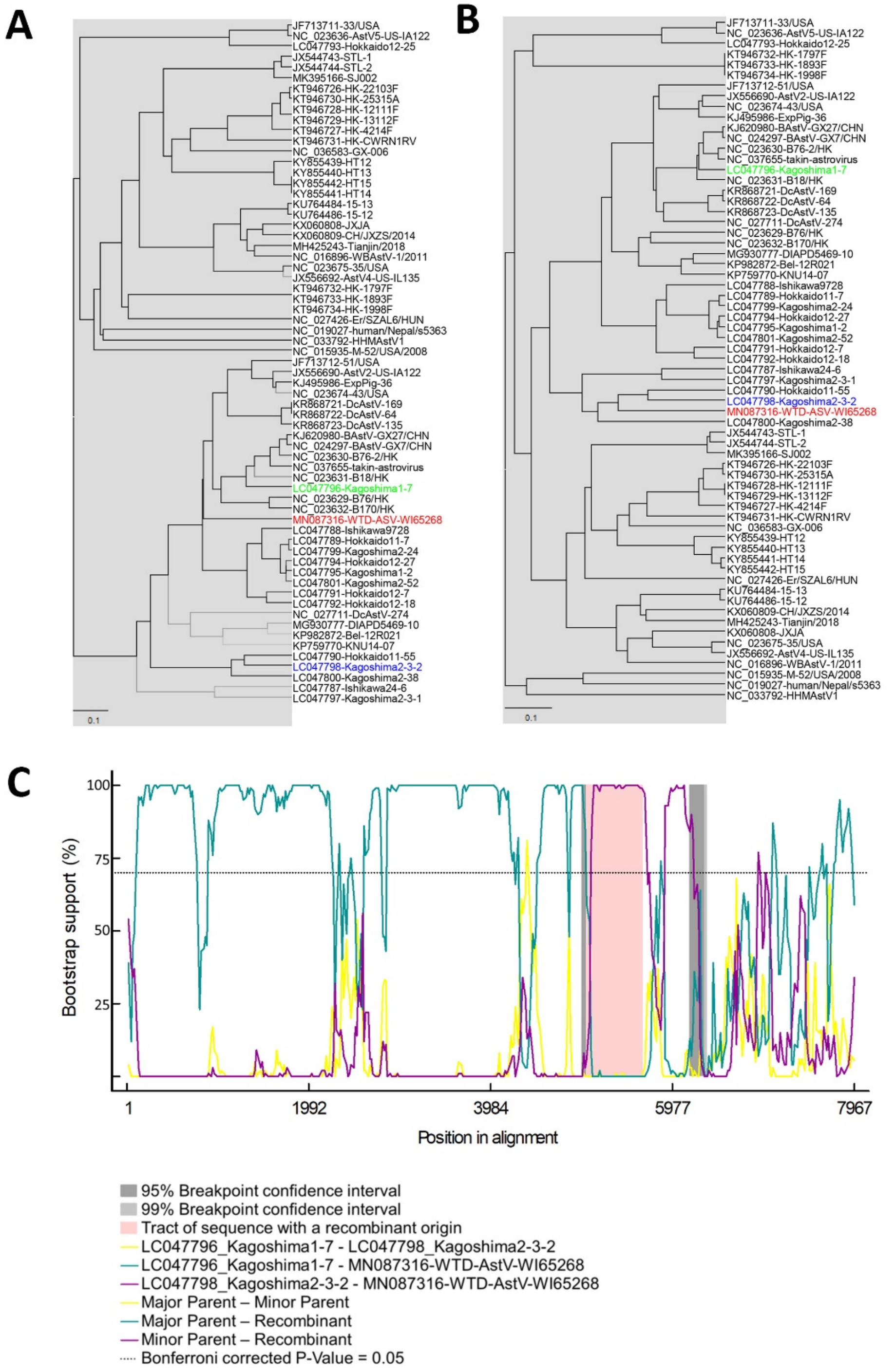

Appendix Figure 4. Recombination analysis of the nucleotide sequence of WTD-AstV-WI65268 using RDP v.5 software. A and B) Two trees of recombinant WTD-AstV-WI65268. Red, green, and blue color shade are used to label the recombinant, major, and minor parent strains on each tree, respectively. C) Bootscan plot. Turquoise blue lines are Major Parent-Recombinant; purple lines are Minor Parent-Recombinant, yellow lines are Major Parent-Minor Parent.
